# Identification of interdependent variables that influence coreceptor switch in R5 SHIV_SF162P3N_-infected macaques

**DOI:** 10.1186/1742-4690-9-106

**Published:** 2012-12-13

**Authors:** Ke Zhuang, Andres Finzi, Jonathan Toma, Arne Frantzell, Wei Huang, Joseph Sodroski, Cecilia Cheng-Mayer

**Affiliations:** 1 Aaron Diamond AIDS Research Center, New York, NY, USA; 2 Centre de Recherche du CHUM, Université de Montréal, Montreal, Quebec, Canada; 3 Monogram Biosciences, Inc, South San Francisco, CA, USA; 4 Dana Faber Cancer Institute, Harvard Medical School, Boston, MA, USA

**Keywords:** R5 SHIV, Coreceptor switch, CD4 binding, Macrophage infection

## Abstract

**Background:**

We previously reported that adoption of an “open” envelope glycoprotein (Env) to expose the CD4 binding site for efficient receptor binding and infection of cell targets such as macrophages that express low levels of the receptor represents an early event in the process of coreceptor switch in two rapidly progressing (RP) R5 SHIV_SF162P3N_-infected rhesus macaques, releasing or reducing Env structural constraints that have been suggested to limit the pathways available for a change in coreceptor preference. Here we extended these studies to two additional RP monkeys with coreceptor switch and three without to confirm and identify additional factors that facilitated the process of phenotypic conversion.

**Results:**

We found that regardless of coreceptor switching, R5 viruses in SHIV_SF162P3N_-infected RP macaques evolved over time to infect macrophages more efficiently; this was accompanied by increased sCD4 sensitivity, with structural changes in the CD4 binding site, the V3 loop and/or the fusion domain of their Envs that are suggestive of better CD4 contact, CCR5 usage and/or virus fusion. However, sCD4-sensitive variants with improved CD4 binding were observed only in RPs with coreceptor switch. Furthermore, cumulative viral load was higher in RPs with than in those without phenotypic switch, with the latter maintaining a longer period of seroconversion.

**Conclusions:**

Our data suggest that the increased virus replication in the RPs with R5-to-X4 conversion increased the rate of virus evolution and reduction in the availability of target cells with optimal CD4 expression heightened the competition for binding to the receptor. In the absence of immunological restrictions, variants that adopt an “open” Env to expose the CD4 binding site for better CD4 use are selected, allowing structural changes that confer CXCR4-use to be manifested. Viral load, change in target cell population during the course of infection and host immune response therefore are interdependent variables that influence R5 virus evolution and coreceptor switch in SHIV_SF162P3N_-infected rhesus macaques. Because an "open" Env conformation also renders the virus more susceptible to antibody neutralization, our findings help to explain the infrequent and late appearance of X4 virus in HIV-1 infection when the immune system deteriorates.

## Background

Entry of the human immunodeficiency virus (HIV) is initiated by binding of the gp120 surface subunit of the viral envelope protein (Env) to the cellular receptor CD4 and either the CCR5 (R5 viruses) or CXCR4 (X4 viruses) coreceptor on the target cell [1]. Most HIV-1 transmissions result in a predominantly R5 virus infection [2-4]. With time, X4 variants arise and coexist with R5 variants in ~50% of subtype B infected individuals, and their emergence is associated with rapid CD4+ T cell loss and disease progression [5,6]. The determinants of phenotypic change from R5 to X4 map largely to the V3 region of the exterior envelope glycoprotein, gp120 [7], and can be inferred by analysis of the amino acid sequence of this region [8]. The underlying factors for virus coreceptor switch late in infection however remain uncertain, but several hypotheses that include high viral load and evolutionary rate, changes in target cell populations during the course of infection and/or differential immune recognition of X4 and R5 viruses have been proposed [9,10]. Because the presence of X4 virus is associated with poorer clinical prognosis and is a major limitation to the clinical use of CCR5 inhibitors [11-15], a better understanding of the pathway and selective pressures for the emergence of X4 virus should provide important insights into HIV-1 pathogenesis and treatment.

We recently reported coreceptor switch in macaques infected intravenously (iv), intrarectally (ir) or intravaginally (ivag) with the late R5 SHIV_SF162P3N_ isolate [16-18]. The majority of infected macaques in which X4 virus emerged are rapid progressors (RP), with a clinical course that is characterized by persistent high levels of virus replication, early onset of clinical disease and undetectable or transient antiviral antibody titers that usually wane within 3-4 weeks of virus inoculation. Nevertheless, we showed that the genetic requirements for coreceptor switch in SHIV_SF162P3N_-infected RPs overlapped with those reported in humans that developed neutralizing antibodies [8,16,17,19,20], and transitioned similarly through dual-tropic intermediates with reduced replicative capacity and less efficient coreceptor use [21-24]. Furthermore, consistent with findings in HIV-1 infected patients [25-27], the appearance of X4 virus follows rather than precedes the initial decline of CD4+ T cells in the infected macaques. Since the newly emerging CXCR4-using viruses in both hosts are highly sensitive to neutralization with antibodies directed against the CD4 binding site [16,17,28,29], these observations suggest that immunological impairment provides a selective advantage for emergence and expansion of X4 virus. Thus, the mechanistic pathways and selection factors underlying phenotypic conversion are likely similar in some HIV-1 infected patients and in SHIV_SF162P3N_-infected RP monkeys.

For these reasons, we conducted a study to explore the mechanistic basis and blockade(s) for virus coreceptor switch in two R5 SHIV_SF162P3N_-infected RPs [30]. We found that R5 viruses evolved over time in these two macaques to become increasingly sensitive to sCD4, indicative of an “open” envelope conformation that exposes the CD4 binding site and improves receptor binding. Indeed, we observed that the increase in sCD4 sensitivity of the evolving R5 viruses correlates with the ability of their Envs to bind CD4 more efficiently as well as to mediate infection of cell targets that express low levels of the receptor. Furthermore, major antigenic changes in Env gp160 of the R5 viruses, including changes in the V3 loop that are important for coreceptor engagement, were seen near and at the time of coreceptor switch, consistent with global changes in Env conformation and structural plasticity that facilitate the remodeling needed to expand or switch to CXCR4 usage. These findings led us to propose that adoption of an "open" Env to expose the CD4 binding site for efficient CD4 binding and infection of CD4^low^ cells represents an early event in the process of coreceptor switch, releasing or reducing Env structural constraints that have been suggested to limit the mutational pathways available for a change in coreceptor preference. Because these studies were limited with respect to the number of animals, and not all R5 SHIV_SF162P3N_-infected RP macaques exhibited coreceptor switch, the present work is conducted in two additional RPs with and three without tropism switch to verify and extend our earlier observations, and to identify additional factors that facilitated the process of R5-to-X4 conversion.

## Results

### R5 viruses evolve in RPs with coreceptor switch to infect primary macrophages more efficiently by improving their CD4 binding

**Figure 1:**
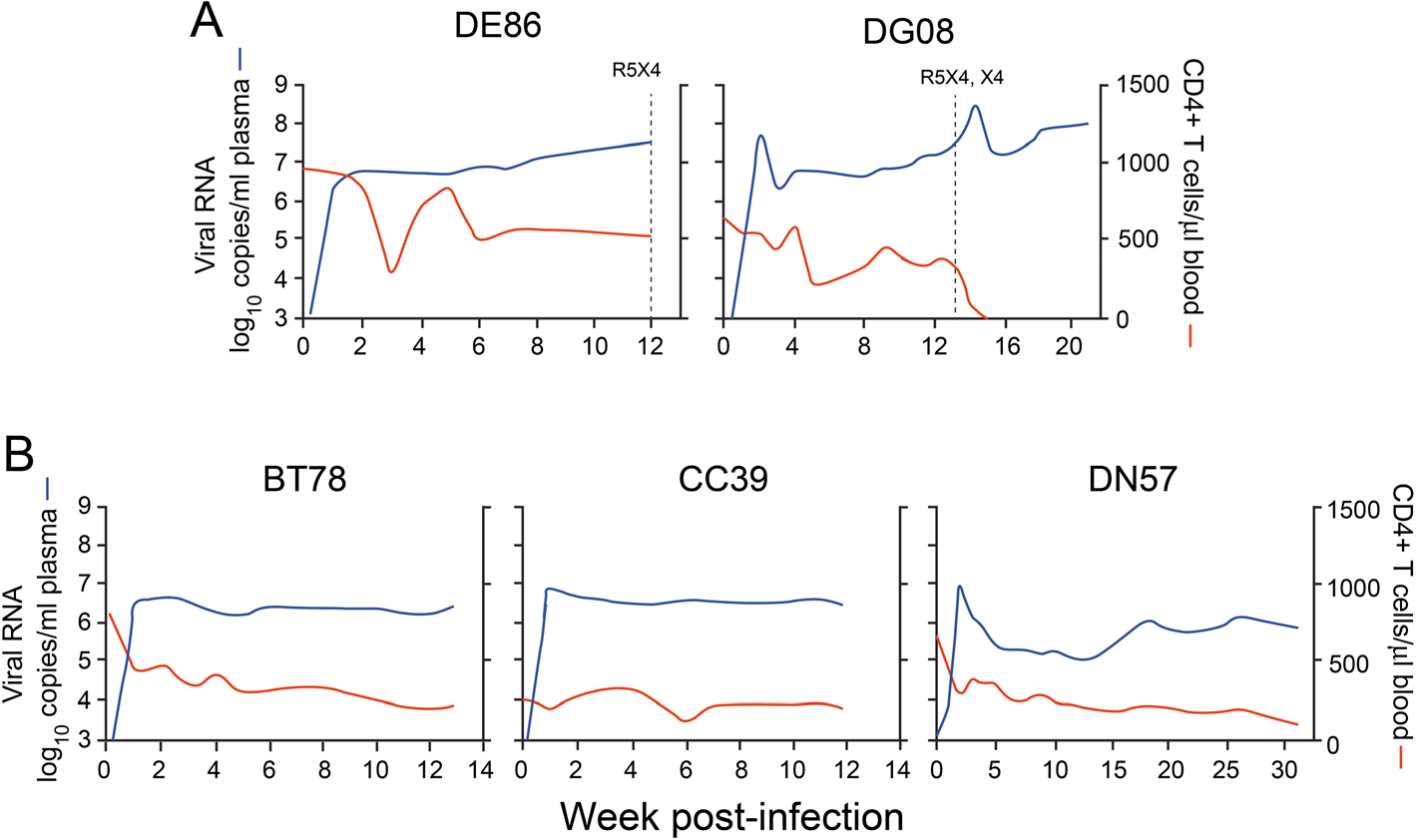
**Viral load and CD4+ T cell count in SHIV**_**SF162P3N**_**–infected macaques with (A) and without (B) coreceptor switch.** (**A**) Macaque DE86 was infected by the intravenous route and DG08 was inoculated intrarectally. Dashed line designates the time of dual-tropic and X4 virus emergence in these animals. (**B**) Macaques BT78, CC39 were infected intravenously while DN57 was challenged intrarectally. Time to euthanasia for the five RPs is: w12 (DE86), w20 (DG08), w13 (BT78), w12 (CC39) and w30 (DN57).

To lend further support to our proposed mechanistic model that exposure of the CD4 binding site for improved CD4 binding and infection of CD4^low^ cells is an early step in coreceptor switching, we generated viruses pseudotyped with CCR5-using Envs amplified over time from two additional SHIV_SF162P3N_-infected RPs with coreceptor switch (DE86, DG08). In addition to clinical indicators such as CD4 T cell loss in the periphery and lymph nodes, coreceptor switch in our studies is defined genotypically and phenotypically by envelope gp120 V3 sequence analysis in combination with an assessment of the ability of viruses recovered from blood and nodes of the infected animals at end-stage disease to utilize CXCR4. We determined the sensitivity of the evolving viruses to sCD4 and the CCR5 inhibitor PSC-RANTES as indirect measurements of their CD4 and CCR5 utilization efficiencies, respectively [31,32]. The ability of the viruses to infect primary macrophages that express low levels of the CD4 receptor and their gp120s to bind CD4-Ig was also examined. High and sustained levels of virus replication were seen in both rhesus monkeys, with progression to disease within 30 weeks post-infection (wpi) (Figure 1A). Two independent systemic R5-to-X4 switch events were identified in DG08 seven weeks prior to euthanasia at 20 wpi [17], with one in DE86 that is localized to the lymph node at the time of necropsy (12 wpi) (unpublished data; [33]). Blood CD4+ T cell count fluctuated in DE86, but declined in DG08, with precipitous loss towards end-stage disease. DG08 failed to mount a detectable anti-SHIV antibody response while the response in DE86 was transient, waning at 4 wpi (Table 1). Full-length gp160 Envs were derived by bulk PCR amplification from plasma collected at 2, 4, 5, 8 and 12 wpi for DE86, and at 2, 4, 8, 11, 12 13, 14, 16 and 20 wpi for DG08. Three or more CCR5-using Env clones per time point were analyzed.

**Table 1:** **Anti-SHIV binding antibody in R5 SHIV**_**SF162P3N**_**-infected RP macaques**

**Week post-infection**	**With coreceptor switch**				**Without coreceptor switch**		
	**BR24**	**CA28**	**DE86**	**DG08**	**BT78**	**CC39**	**DN57**
0	-	-	-	-	-	-	-
1	-	+	-	-	-	+	-
2	+	+	+	-	+	-	-
3	+	+	+	-	+	-	+
4	+	-	+	-	+	-	+
5	-		-	-	+		
6		-				-	+
7		+	-	-	+	-	
8	-		-		+		+
9		-				-	+
10				-	+		+
11		-				-	+
12	-		-*		-	-*	
13		-			-*		+
14				-			
15		-*					+
16	-						
17							+
18				-			
19							+
20	-			-*			
24	-						
25							+
28	-*						
30							+*

*Indicates time of euthansia. BR24, CA28, DE86, BT78 and CC39 were infected intravenously, while DG08 and DN57 were infected intrarectally. BR24 and CA28 are RPs from an earlier study [30].

**Figure 2:**
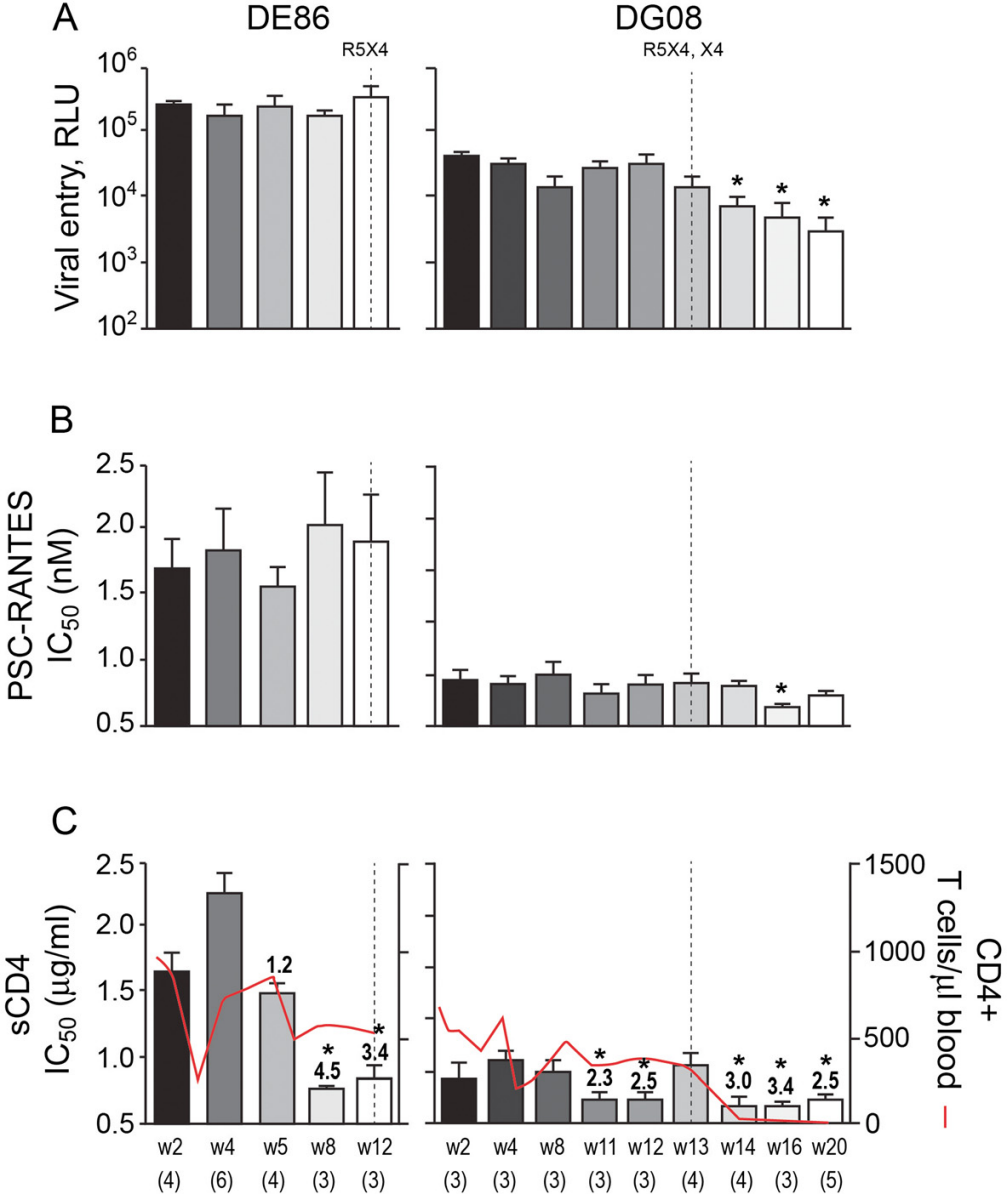
**Entry efficiency, PSC-RANTES and sCD4 sensitivity of R5 viruses evolving over time in DE86 and DG08.** Entry of luciferase reporter viruses expressing CCR5-using envelopes into TZM-bl cells expressed as relative light unit (RLU) (**A**), and susceptibility of the reporter viruses to neutralization with PSC-RANTES (**B**) and sCD4 (**C**) were determined. The dashed vertical line indicates time of tropism switch in DE86 (12 wpi), and DG08 (13 wpi). The numbers in the brackets indicate the number of clones analyzed at each time point. Absolute CD4+ T cell count in the animal over the course of infection is shown in (C) for reference and values above the bars indicate fold increase in sCD4 sensitivity relative to that of the w2 viruses. * P<0.05 (Mann-Whitney *U* test). Data are representative of 2-3 independent experiments (error bars, s.d.).

We found no significant difference in the entry efficiency (Figure 2A) or susceptibility to PSC-RANTES inhibition of R5 viruses evolving over time in DE86 (Figure 2B). In contrast, R5 viruses that evolved following the time of emergence of dual- and X4-tropic viruses in DG08 infected the CD4^hi^CCR5^hi^ TZM-bl cells less efficiently (8.4- and 13-fold reduction in RLU for the w16 and w20 viruses as compared to the early w2-4 viruses respectively), with a 1.5- to 2-fold increase in susceptibility to PSC-RANTES inhibition of the w16 and w20 viruses that is suggestive of less efficient CCR5 usage. These findings of decreased replication and efficacy of CCR5 use with disease progression in DG08 are consistent with results in HIV-1 infected individuals with detectable CXCR4-using variants [6,34,35] and in the two SHIV_SF162P3N_-infected RP macaques with coreceptor switch studied earlier (BR24, CA28) [30]. Moreover, in agreement with our previous data, R5 viruses in both DE86 and DG08 evolved to become increasingly sensitive to inhibition with CD4-IgG2, a tetrameric soluble CD4 (sCD4) construct based on IgG. For both monkeys, the increase in sCD4 sensitivity that preceded the coreceptor switch occurred in the presence of adequate CD4+ T cell numbers (300-500 CD4+ T cells per ul blood; Figure 2C). Compared to the early w2 replicating virus, which required 1.45 μg/ml sCD4 to achieve 50% neutralization (IC_50_), a significant 3-4.5 fold increase in susceptibility was evident for viruses prior to (w8) and at the time of (w12) coreceptor switch in DE86. The w2 replicating virus in DG08 was as sensitive to sCD4 neutralization as the late DE86 viruses (IC_50_ 0.42 μg/ml), but viruses replicating two weeks later were slightly more sCD4 resistant (IC_50_ 0.6 μg/ml). Compared to the w4 viruses, R5 viruses present before (w11, w12) and after (w14, w16, w20) the time of switch in DG08 were also significantly more sCD4 sensitive, with a rebound in sCD4 resistance close to the baseline level seen at the time of switch (w13).

**Figure 3:**
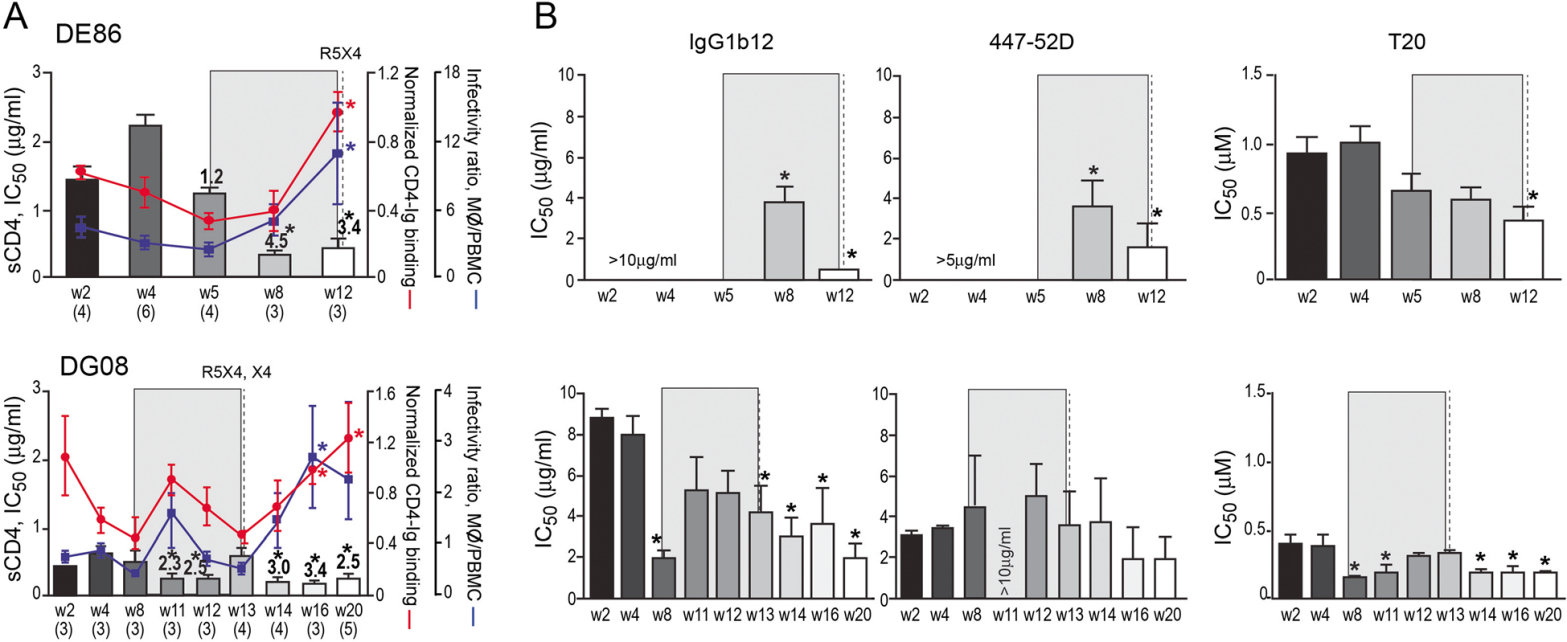
**(A) Relationship between sCD4 sensitivity, CD4-Ig binding and infection of primary macrophages (mΦ) of DE86 and DG08 viruses**. Values above the bars indicate fold increase in sCD4 sensitivity of evolving viruses compared to early viruses, and the vertical dashed line indicates the time of coreceptor switching. sgp120 binding to CD4-Ig was normalized to that of sgp120 binding to polyclonal serum from HIV-1 infected individuals. Infectivity in mΦ that express low levels of CD4 was expressed as a ratio of infectivity in autologous PBMCs that express high levels of CD4 and CCR5. The shaded area highlights the time prior and during coreceptor switch. For sgp120 CD4-Ig binding, data are the means and standard deviations from at least two independent experiments. For infection of macrophages, data are representative of at least 3 independent experiments (error bars, s.d.). * indicates statistically significant differences between the early and the evolving R5 viruses. (**B**) Changes in neutralization sensitivity of R5 viruses evolving over time in macaques DE86 and DG08. Susceptibility of R5 pseudoviruses to neutralization with IgG1b12, 447-52D and T20 was determined. The vertical dashed line indicates the time of coreceptor switching, and the shaded area designates the period of marked envelope conformational changes. Data are representative of at least two independent experiments (error bars, s.d.). * above the bars indicate IC_50_ values that are statistically different between the acute and the evolving R5 viruses, P<0.05 (Mann-Whitney *U* test).

Importantly, and in support of our earlier findings [30], the increase in sCD4 sensitivity of the evolving R5 viruses in DE86 and DG08 was accompanied by a corresponding increase in the binding of the gp120s to CD4-Ig and in infection of primary macrophages that express low amounts of the CD4 receptor (Figure 3A). The exceptions were R5 viruses present one week prior (w12) and at the time of coreceptor switch (w13) in DG08. Despite sCD4 sensitivity that was comparable to the w11 viruses, the w12 viruses in DG08 exhibited diminished ability to infect primary macrophages, with lower gp120/CD4-Ig binding as well. We did not observe any amino acid changes in the CD4 binding site, the Phe43 cavity or the inner domain layers of gp120 that could explain the loss in macrophage infection and CD4 binding of the w12 Envs. This dissociation between sCD4 sensitivity, better CD4 binding and infection of CD4^low^ cells in DG08 prior to the time of switch had previously been observed in BR24 [30], suggesting that mechanism(s) other than exposure of the CD4 binding site (BS) for better CD4 use is conferring sCD4 sensitivity to the w12 viruses. Decreased infection of primary macrophages and receptor binding was also seen for the w13 viruses, but as noted above, there was a 2-fold increase in sCD4 resistance for R5 viruses at this time point.

### Changes in envelope glycoprotein antigenic structure near and at the time of tropism switch

Marked changes in structure or accessibility of the CD4BS and V3 loop of gp120 and the pre-hairpin intermediate of gp41 were observed near the time of switch in our prior study of two RPs [30]. Accordingly, we probed the structure of the evolving R5 viruses in DE86 and DG08 by assessing their susceptibility to neutralization with the anti-CD4BS mAb b12, the anti-V3 loop mAb 447-52D and T20, which binds a triple-stranded coiled coil activated fusion intermediate composed of the N-terminal heptad repeat (HR-1) region of gp41. Results showed that the changes in sCD4 sensitivity and receptor use of the R5 viruses evolving around the time of coreceptor switch in the two macaques coincided with modulations in Env conformations. For DE86, there was a significant increase in b12 and 447-52D susceptibility for viruses close to (w8) and at the time of (w12) coreceptor switch that may be indicative of changes in the structure and/or accessibility of the CD4 and coreceptor binding sites (Figure 3B). A significant increase in T20 sensitivity of the w12 viruses is suggestive of greater exposure or a longer half-life of the gp41 HR-1 groove on the Envs of these viruses. The changes in the antigenic structure of R5 Envs around the time of coreceptor switch in DG08, the macaque that harbored two independent R5-to-X4 evolutionary pathways, were more complex. Compared to the acute viruses, a statistically significant increase in b12 sensitivity was evident at 8 wpi, and remained so except for the w11 and w12 viruses. T20 sensitivity was also significantly increased, with the exception of viruses prior to (w12) and at the time of switch (w13). Furthermore, a dramatic decrease in 447-52D sensitivity was seen for the w11 viruses. Taken together, the data support Env structural changes near and following the time of switch in DE86 and DG08. These changes involve the V3 domain that is important for coreceptor binding and the fusion peptide in gp41 that can modulate coreceptor specificity [36]. The frequent blood samplings in DG08 (weekly before, during and after tropism switch) provided a more dynamic picture of R5 virus evolution in RPs with multiple and concurrent coreceptor-switching events.

### R5 viruses in RPs without coreceptor switch also evolved to infect macrophages more efficiently, but this was not accompanied by an increase in CD4 binding

**Figure 4:**
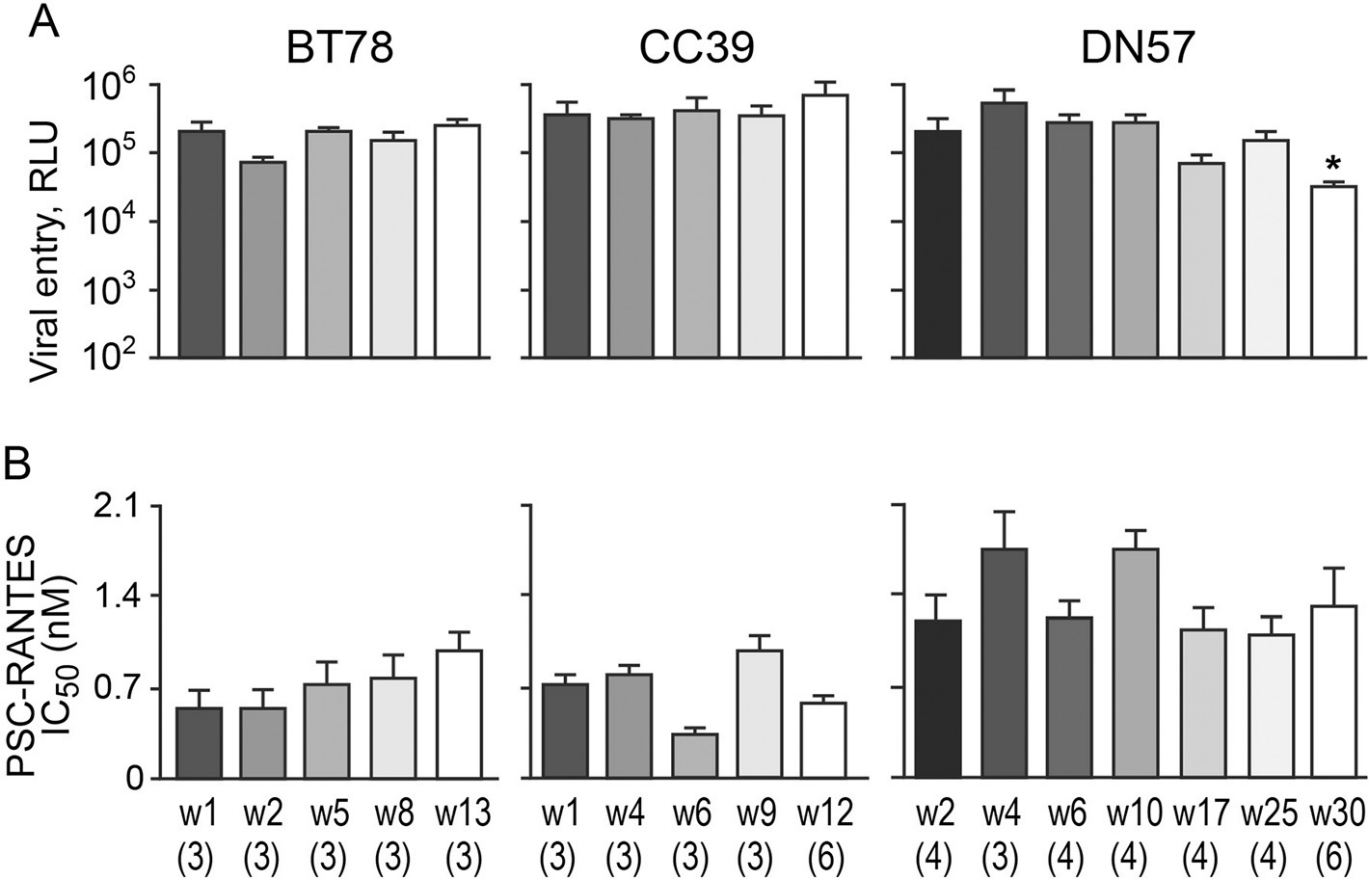
**Entry efficiency, PSC-RANTES and sCD4 sensitivity of R5 viruses evolving over time in BT78, CC39 and DN57.** Entry of luciferase reporter viruses expressing CCR5-using Envs into TZM-bl cells expressed as relative light unit (RLU) (**A**), and susceptibility of the reporter viruses to neutralization with PSC-RANTES (**B**) and sCD4 (**C**) were determined. The numbers in the brackets indicate the number of clones analyzed at each time point and values above the bars indicate fold increase in sCD4 sensitivity relative to that of the w2 viruses. * P<0.05 (Mann-Whitney *U* test). Data are representative of 2-3 independent experiments (error bars, s.d.).

To determine if acquisition of better CD4 binding is a unique characteristic of the R5 viruses in RPs with coreceptor switch, we characterized CCR5-using Envs over time from three SHIV_SF162P3N_-infected RP macaques with no overt signs of coreceptor switch. BT78, CC39 and DN57 progressed to disease at 13, 12 and 30 wpi respectively, with preservation of peripheral and lymphoid CD4+ T cells at the time of euthanasia [17,33](Figure 1B). Weak SHIV-specific binding antibody response could be detected throughout the course of infection in DN57, but was less persistent in BT78 (waned after 10 wpi) and absent in CC39 (Table 1). We derived full-length Envs by bulk PCR amplification of plasma collected at 1, 2, 5, 8 and 13 wpi for BT78, at 1, 4, 6, 9 and 12 wpi for CC39, and at 2, 4, 6, 10, 17, 25 and 30 wpi for DN57. At least three Env clones from each time point were assessed for their ability to mediate entry and PSC-RANTES susceptibility. Results showed that Envs amplified over time from BT78 and CC39 mediated comparable entry into TZM-bl cells, but the late viruses in DN57 (w17, 25 and 30) showed a 2- to 7-fold reduction in entry efficiency compared to the acute w2 viruses (Figures 4A). PSC-RANTES sensitivity fluctuated for the evolving R5 viruses in CC39 and DN57, but a trend towards resistance was seen in BT78 (Figure 4B). The difference in PSC-RANTES susceptibility between the early w2 and end-stage w13 viruses in BT78 approaches significance (p=0.06), consistent with reports that the late R5 viruses in HIV-1 infected individuals without detectable X4 viruses use the CCR5 coreceptor better [6,37-41].

**Figure 5:**
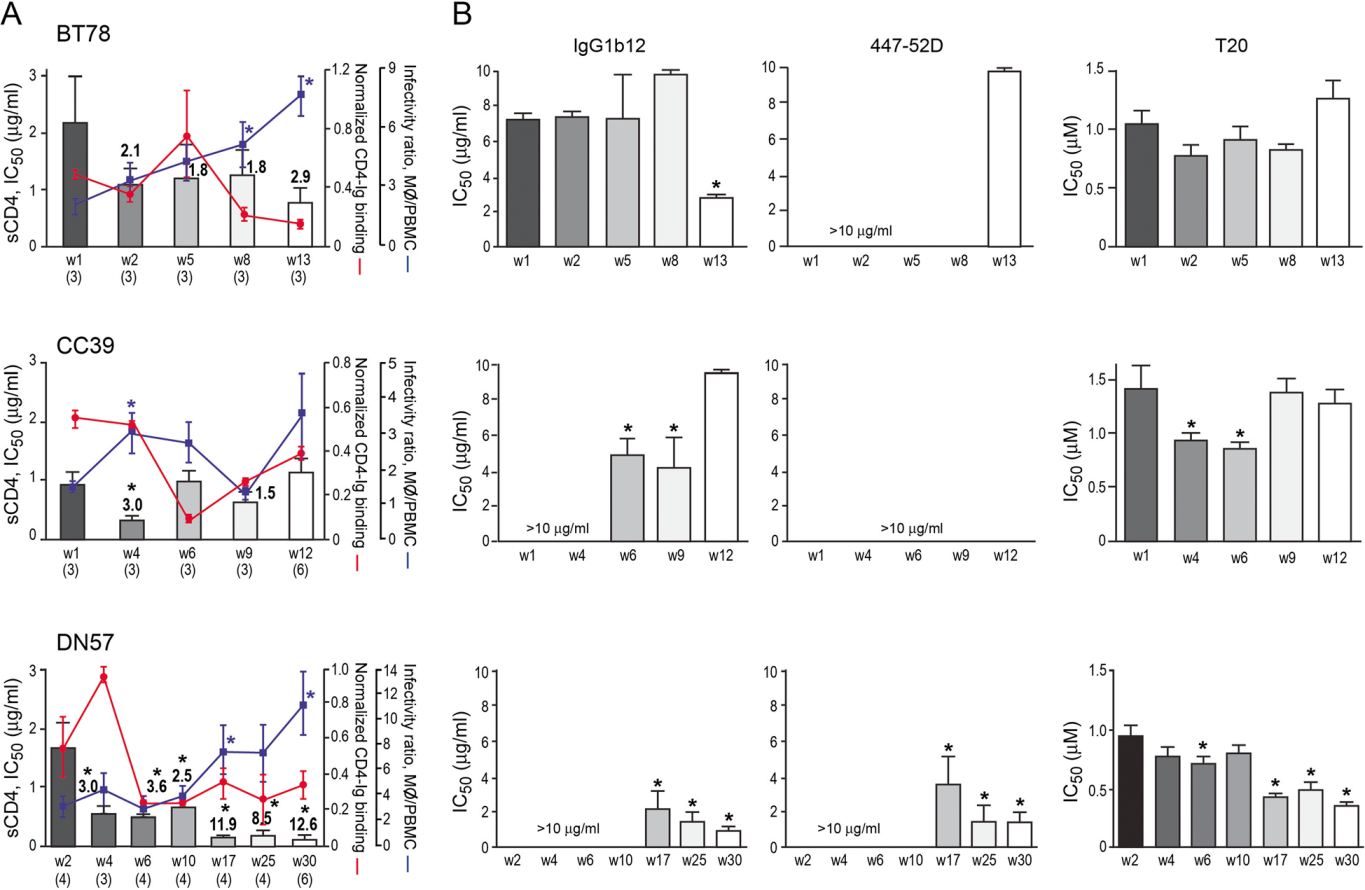
**Enhanced macrophage infection accompanied by Env structural changes in R5 viruses evolving over time in macaques BT78, CC39 and DN57.** The relationship between sCD4 sensitivity, binding of sgp120 to CD4-Ig, infectivity of primary macrophages (mΦ) (**A**), and neutralization susceptibility (**B**) of pseudoviruses bearing CCR5-using Envs amplified over time from BT78, CC39 and DN57 is shown. Data are representative of at least two independent experiments (error bars, s.d.). * above bars indicate differences in sCD4 sensitivity, CD4-Ig binding and susceptibility to agents and antibodies between the acute (w2) and the evolving R5 viruses that are statistically significant, * P<0.05 (Mann-Whitney *U* test).

sCD4 sensitivity, gp120/CD4-IgG binding and infection of primary macrophages of the evolving R5 viruses in the three RPs without evidence of coreceptor switch were then determined (Figure 5). Increase in sCD4 sensitivity was seen for R5 viruses evolving over time in BT78 and DN57, but was transient in CC39. The increase was ~3-fold for the end-stage (w13) viruses relative to the early (w2) viruses in BT78, but was significant in DN57 (Figure 5A). Compared to the acute (w2) viruses, a 3-fold increase in sCD4 sensitivity was seen as early as 4 wpi, with further increases (8-13 fold) for the late viruses (w17, 25 and w30). Notably, an increase in the ability of the evolving viruses to infect macrophages was seen in all three RPs. The increase was significant for the w4 viruses in CC39 as compared to the w1 viruses (p<0.05), with differences that approach significance for the w9 and w12 viruses in this animal (p=0.07 and p=0.09, respectively). For BT78 and DN57, the difference in the ability to infect CD4^low^ cells between the acute and late viruses was significant. Importantly, however, there was no corresponding increase in the ability of the gp120s of the evolving R5 viruses in these animals to bind CD4-Ig that would indicate increase accessibility of the CD4 binding site (Figure 5A). We recognize that CD4 binding was measured using monomeric gp120 and may differ from that of trimeric gp120. Nevertheless, because the comparison is between CD4 binding of monomeric gp120s evolving over time in macaques with and without coreceptor switch, the finding that increased gp120/CD4 binding is observed only in animals with tropism switch is still of interest. Thus, we conclude that while the selective pressure for infection of alternative target cells with lower receptor expression levels is present in RPs with and without coreceptor switch, R5 viruses in macaques that did not switch evolved to be more sCD4 sensitive and infected CD4^low^ cells more efficiently via a mechanism other than adoption of an “open” Env conformation to expose their CD4BS for better receptor binding.

### R5 viruses in RPs without coreceptor switch employ a compensatory mechanism for poor CD4 binding in infection of macrophages and in sCD4 susceptibility

Besides exposure of the CD4BS for efficient CD4 binding, increase sensitivity to sCD4 neutralization and infection of CD4^low^ cells may mean that the conformational changes induced by CD4 binding are altered, that the virus has acquired additional contacts with CD4, or the interaction with CCR5 is improved [42-48]. The greater resistance to PSC-RANTES inhibition of the late viruses (w13) in BT78 is suggestive of better use of the CCR5 coreceptor, and changes in gp41 HR1 has been associated with SIV macrophage tropism [49,50]. To further understand the mechanistic basis for increase sCD4 sensitivity and infection of CD4^low^ cells of the evolving R5 viruses in BT78 and DN57, their antigenic structure was examined. We found that the late viruses in BT78 (w13) and DN57 (w17, 25 and 30) were significantly more sensitive to IgGb12 (>3–fold), suggesting alteration in the structure of the CD4BS of these viruses that could provide additional CD4 contacts. Changes in susceptibility to the anti-V3 mAb 447-52D were also seen for BT78 and DN57, with significant increase in sensitivity for the late viruses in DN57 that may be indicative of the exposure of the V3 loop. Moreover, the late viruses in DN57 were significantly more sensitive to T20, which has been correlated with enhanced exposure of the HR1 fusion domain in gp41. Similar changes were seen for in CC39, with significant increases in IgGb12 and T20 sensitivity seen for the w6 and w9 viruses. Increase CD4 contact, exposure of the V3 loop and HR1 groove for virus fusion therefore could have compensated for the poor CD4 binding of the late viruses in the three macaques in conferring sCD4 sensitivity and in adapting to less-than-optimal CD4 molecules for entry into primary macrophages.

### High-level replication is a strong predictor for R5-to-X4 virus evolution in RPs

**Figure 6:**
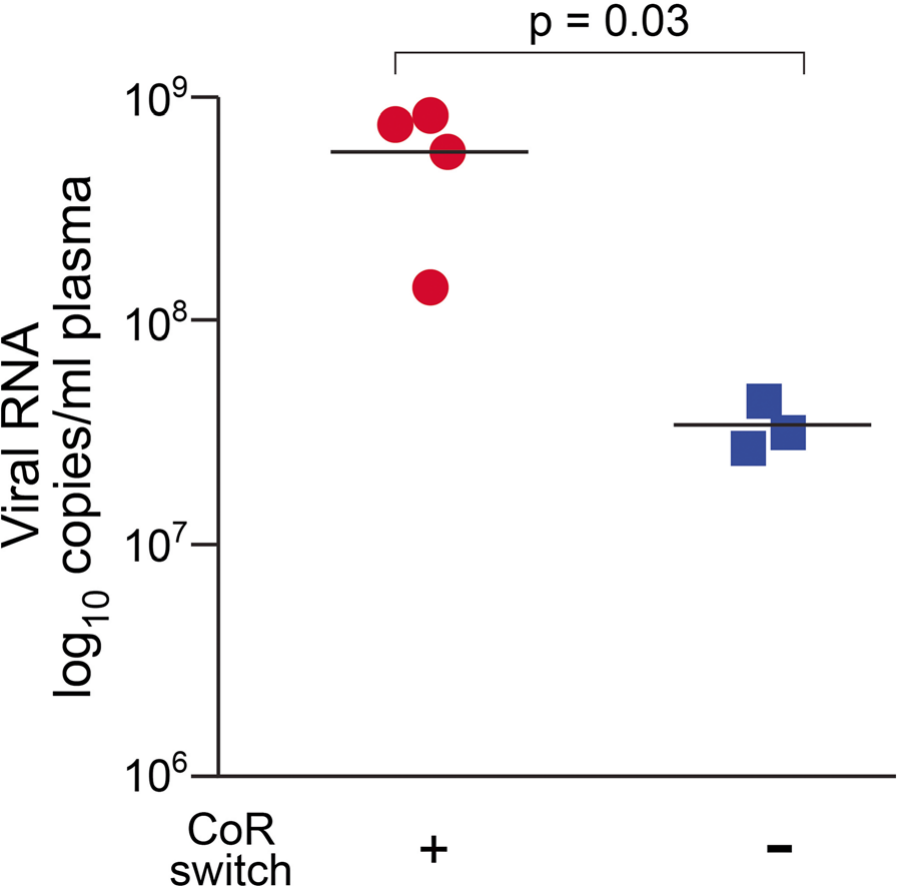
**Comparison of cumulative viral load in R5 SHIV**_**SF162P3N**_**-infected RPs with (n=4) and without (n=3) coreceptor switch.** Cumulative log_10_ RNA copies/ml of plasma viremia up to the time of euthanasia were compared using unpaired t tests. Time to euthanasia for the four RPs with coreceptor switch is: w24 (BR24), w15 (CA28), w12 (DE86), w20 (DG08), and for the three RPs without coreceptor switch is: w13 (BT78), w12 (CC39) and w30 (DN57). *P* value of <0.05 was considered significant.

The finding that not all SHIV_SF162P3N_ –infected RP macaques adapt to use low levels of CD4 through adoption of an “open” Env to enhance CD4 binding, even though such an Env configuration would have relieved the structural constraints on change in coreceptor preference, suggests that there is no obligatory selection pressure for the virus to use CXCR4. It also implies that the absence or diminution of antibody-driven pressure by itself is insufficient to drive coreceptor switching, and is consistent with our report that depletion of B cells to abrogate or diminish antiviral antibody responses prior to infection of rhesus macaques with SHIV_SF162P3N_ did not promote tropism switch [51]. In a number of cross-section studies of HIV-1-infected individuals, high viral load was shown to be the strongest predictor of R5-to-X4 evolution [37,52-59]. To determine if high viral load is also a predictor to R5-to-X4 evolution in RP macaques, we compared cumulative viral loads up to the time of euthanasia in R5 SHIV_SF162P3N_-infected RPs with (n=4) and without (n=3) coreceptor switch studied here and previously [30]. We found that cumulative viral load was significantly higher in monkeys with tropism switch than in those without, with a p value of 0.03 (Figure 6). Thus, similar to HIV-1 infection in humans, high virus replication is associated with the evolution and establishment of X4 variants in R5 SHIV-infected macaques.

## Discussion

In this study, we confirmed adoption of an "open" Env to expose the CD4 binding site for efficient CD4 binding and infection of primary macrophages in two additional RPs with coreceptor switch. Moreover, changes in neutralizing antibody and T20 sensitivity around the time of X4 virus emergence are consistent with a more responsive quaternary configuration of the viral envelope spike. In agreement with reports in HIV-1-infected individuals, high virus replication is a strong predictor for R5-to-X4 conversion in SHIV_SF162P3N_-infected macaques. A certain level of viremia may provide the conditions optimal for coreceptor switching or, alternatively, the Env changes required for coreceptor switching may predispose to higher levels of virus replication. Adoption of an "open" Env conformation that confers structural flexibility to accommodate mutational changes, and high levels of virus replication to overcome the genetic hurdles for R5-to-X4 evolution therefore contribute to the coreceptor switch in nonhuman primates. Since an "open" Env conformation also renders the virus more susceptible to antibody neutralization, our findings help to explain the infrequent and late appearance of X4 virus in HIV-1 infection when the immune system deteriorates.

Diminished entry efficiency of the evolving R5 viruses following the time of switch, with increase in PSC-RANTES sensitivity that is suggestive of poorer CCR5 usage was seen in DG08 studied here (Figure 2) and in BR24 and CA28 reported earlier [30]. Interestingly, these are the three R5 SHIV_SF162P3N_-infected RPs that harbored pure X4 virus populations. Decrease in the efficiency of CCR5 usage had also been reported in HIV-1 infected individuals with detectable CXCR4-using variants [6,34,35], suggestive of a decline in R5 fitness. HIV-1 and SHIV viruses with a pure X4 phenotype generally have higher replication kinetics in vitro [60-64] and in vivo [65-68]. Coupled with the broader cellular host range of X4 viruses in vivo [69,70], they are likely to out-compete R5 viruses once established. The decrease in the selective pressure for R5 viruses to maintain optimal replicative capacity and fitness with X4 emergence therefore, could be a reason for the decrease in entry efficiency and CCR5 utilization of R5 viruses in SHIV-infected macaques with pure X4 viruses. Nevertheless, R5 variants persist following the appearance of X4 viruses in these animals, perhaps because of their distinct target cell range [69,70].

Transmitted/founder viruses in acute and early HIV-1 infection have been reported to be sCD4 resistant and to replicate poorly in monocyte-derived macrophages that express low levels of the CD4 receptor [3,71,72]. Neutralization resistance in vitro is often accompanied by an increase in HIV-1 dependence on CD4 for entry [73-75]. Indeed, founder viruses were shown to require high levels of CD4 for entry [76], which may explain their deficiency in infecting CD4^low^ cells. Consistent with these findings in humans, viruses that are sCD4 resistant and infected primary macrophages less efficiently predominated during acute infection in R5 SHIV_SF162P3N_-infected RP macaques DE86, BT78, CC39 and DN57, and in the two RPs we previously studied [30], regardless of coreceptor switching. The exception was DG08, where the acute (w2) viruses were observed to be sCD4 sensitive but did not infect macrophages efficiently. This however, was rapidly replaced two weeks later by variants that are more resistant to sCD4, reinforcing the view that these viruses are selected for during primary infection. As this was observed despite intravenous inoculation (BR24, CA28, DE86, BT78, CC39) which circumvented mucosal barriers, and in macaques that failed to mount a strong humoral or cellular immune response, these factors are unlikely to be playing a role in the early expansion of sCD4-resistant viruses. We speculate that the abundance of target cells with high levels of cell surface CD4 expression in the new hosts maybe an underlying determinant for the expansion and propagation of viruses that are more dependent on CD4 for entry during acute SHIV_SF162P3N_ infection in macaques and HIV-1 infection in humans. Further research on the importance of CD4 receptor density in HIV infection as well as the kinetics of virus replication in infected cells may help to understand the selective forces that govern the biological phenotype of HIV-1 transmitted founder viruses.

Enhanced infection of primary macrophages was seen in the RP macaques at end-stage disease irrespective of coreceptor switch, indicative of a strong selective pressure to replicate in cells with lower CD4 cell-surface expression levels over the infection course. Adaptation to macrophages has also be observed in SIV-infected RP macaques [77], and isolates from late in disease of HIV-1 infected individuals have been reported to be more macrophage-tropic compared to those from early in infection [78-80], suggestive of similar selection pressure in humans and rhesus monkeys. Presumably, as discussed above, ongoing virus replication in both hosts leads to loss in target cells (e.g., memory T cells) with high receptor expression levels, putting pressure on the virus to change so that it can use CD4 more efficiently to infect alternate CCR5+ cells such as macrophages that express low amounts of receptor. Infection-associated immune activation may also decrease receptor density, since CD4 is down-regulated with T-cell activation [81-83]. In RPs with coreceptor switch (BR24, CA28, DE86, DG08), infection of CD4^low^ cells was achieved by adoption of a less constrained and more “open” Env conformation that exposes the CD4 binding site for better CD4 binding, allowing flexibility in accommodating the structural remodeling needed for the change in coreceptor preference. The V3 loop mutations that confer CXCR4 usage in R5 SHIV_SF162P3N_-infected RP monkeys and in HIV-1 infected individuals also render the virus sCD4 sensitive, consistent with the notion that improved CD4 binding is a necessary prerequisite for acquisition of CXCR4 use [23,84,85]. In RP macaques that did not switch, R5 viruses appeared to have evolved to compensate for inadequate CD4 binding by improving CCR5 usage and/or the requisite post-binding conformational changes for virus entry in response to non-optimal CD4. It is possible that given time, these latter macaques (e.g. BT78, CC39) could also adopt an “open” Env conformation to facilitate CD4 binding and infection of primary macrophages, setting the stage to tropism switch. Alternatively, the presence of host immune response may be a hindering factor. This is best illustrated in DN57, which, contrary to the other RP monkeys, mounted a persistent albeit weak anti-SHIV antibody response. R5 viruses evolved in this macaque to be highly sCD4 sensitive, with IC_50_ values comparable to those seen in DE86, suggestive of a similar strong selective pressure for better CD4 usage. The evolving R5 viruses in DN57 also infected macrophages efficiently, but this was not accompanied by a corresponding increase in CD4 binding that is indicative of exposure of the receptor binding site. Rather, the Envs in DN57 evolved to adapt to less-than-optimal CD4 density by changes in receptor and/or coreceptor contact, or by acquiring a higher propensity for proceeding to fusion. Thus, although the pressure to infect cell targets that express low levels of CD4 is present in SHIV_SF162P3N_-infected RP macaques regardless of coreceptor switch, the mechanisms employed to overcome this selective pressure vary depending on other conditions in the host.

## Conclusions

In summary, our studies revealed an ordered process of phenotypic switch that is now recapitulated in four R5 SHIV_SF162P3N_-infected RPs. The data reinforce the view that an interplay between positive (e.g., magnitude of persistent virus replication, evolutionary rate, change in target cell population) and negative (e.g., host immune response) selection forces that differ between individual hosts and at different stages of the infection course governs how R5 viruses respond to the changing environment and shape the frequency of expansion or switch to CXCR4 use. Greater viral burden translates into a faster reduction in the target cell population, higher mutation frequencies of the virus populations and increased chances of X4 evolution. In the absence of immunological constraints in the RPs, and with the conferred selective advantage of expanding cellular host range, X4-associated changes become fixed and these viruses rapidly spread. Since an "open" Env conformation for improved CD4 binding and infection of CD4^low^ cells also renders the virus more susceptible to antibody neutralization, our findings may explain the infrequent and late appearance of X4 virus in typical progressors of HIV-1 infection.

## Methods

### Ethics statement

This work used blood from SHIV infected macaques housed at the Tulane National Primate Research Center (TNPRC) in accordance with the Animal Welfare Act and Guide for the Care and Use of Laboratory Animals. TNPRC is accredited by the Association and Assessment and Accreditation of Laboratory Animal Care (AAALAC #00594). The OLAW animal welfare assurance number for TNPRC is A4499-01 and the USDA registration number is 72-R-002. All procedures were performed on anesthetized animals and post-operative analgesics were administered as needed in accordance with the Tulane IACUC approval.

### Cells

293T cells and Hela TZM-bl cells expressing CD4, CCR5 and CXCR4 and containing integrated reporter genes for firefly luciferase and β-galactosidase under control of the HIV-1 LTR [86] were maintained in DMEM supplemented with 10% fetal bovine serum (FCS), 100 U/ml penicillin, 100 μg/ml streptomycin and 2 mM L-glutamine. Human peripheral mononuclear cells (PBMCs) were prepared by Ficoll gradient centrifugation, stimulated with phytohemagglutinin (PHA, 3 μg/ml; Sigma, St. Louis, MO) in RPMI medium containing 10% FCS, penicillin, streptomycin, L-glutamine and 20 U/ml interleukin-2 (Norvatis, Emeryville, CA). Monocytes were enriched by centrifugation of PBMCs through a 40% percoll cushion followed by plastic adherence, and cultured in RPMI 1640 medium supplemented with 10% FCS and 5% human AB serum for 5-7 days to allow for differentiation into macrophages [87].

### Detection of antiviral humoral response

SHIV-specific antibodies in serum samples were measured by enzyme-linked immunosorbent assay (ELISA) according to the manufacturer’s instructions (GS HIV-1/HIV-2 *PLUS O* EIA; Bio-Rad, Redmond, WA). This assay detects antibodies to HIV-1 gp160 and p24, and to the immunodominant region of the transmembrane glycoprotein gp36 of HIV-2. Optical density values at a 1:10 serum dilution that are three times above the cutoff value are considered positive.

### Plasmid constructs and pseudotyped virus production

For expression of envelope glycoproteins (Env), viral RNA was prepared from 300-500 μl plasma using a commercially available RNA extraction kit (Qiagen, Chatsworth, CA) followed by reverse-transcription (RT) with Superscript III RT (Invitrogen, Carlsbad, CA) and random hexamer primers (Amersham Pharmacia, Piscataway, NJ). Full-length gp160 coding sequences were amplified from bulk RT products with primers SH43 (5^′^-AAGACAGAATTCATGAGAGTGAAGGGGATCAGGAAG-3^′^) and SH44 (5^′^-AGAGAGGGATCCTTATAGCAAAGCCCTTTCAAAGCCCT-3^′^), subcloned into the pCAGGS vector and sequenced for verification. To generate luciferase reporter viruses capable of only a single round of replication, envelope *trans*-complementation assay was used as previously described [88]. Briefly, Env expression plasmid and the NL4.3LucE-R+ vector were cotransfected with polyethylenimine (PEI, Polyscience, Warrington, PA) into 2.5 × 10^6^ 293T cells plated in a 100-mm plate. Cell culture supernatants were harvested 72 hours later, filtered through 0.45-μm filters, and stored at -70°C in 1-ml aliquots. Pseudotyped viruses were quantified for p24 Gag content (Beckman Coulter, Fullerton, CA).

### Virus infectivity

For assessment of Env functionality and entry efficiency, 7 × 10^3^ TZM-bl cells were seeded in 96-well plates 24 hours before use and infected, in triplicates, with 2 ng p24 Gag equivalent of the indicated pseudotyped viruses. Infected cells were cultured for 72 h at 37°C, at which time the cells were harvested, lysed and processed for luciferase activity according to the manufacturer’s instructions (Luciferase Assay System; Promega, Madison, WI). Entry, as quantified by luciferase activity, was measured with an MLX microtiter plate luminometer (Dynex Technologies, Inc., Chantilly, VA). For infection of primary cells, 10^5^ and 10^6^ cells of human PBMCs and macrophage respectively were infected in duplicates with 5 ng p24 Gag equivalent of the indicated pseudotyped viruses in each well of a 96-well plate. Infected cultures were harvested 72 hours later and processed for luciferase activity. To control for differences in Env entry efficiencies, infectivity in macrophages was expressed as a ratio of the infectivity for these cells compared to the infectivity in PBMCs from the same donor.

### Receptor and coreceptor usage efficiency

For assessment of receptor usage efficiency, 2 ng p24 equivalent of the indicated pseudotyped viruses in 50 μl were incubated with equal 4-fold serial dilution volumes of CD4-IgG2 fusion protein (sCD4; PRO 542, Progenics Pharmaceuticals, Tarrytown, NY) for 1 h at 37°C and then added to cells, in duplicate wells, for an additional 2 hours at 37°C. 100 μl of medium was then added to each well and the virus-protein cultures maintained for 72 hours. Control cultures received virus in the absence of sCD4. At the end of the culture period, the cells were lysed and processed for β-galactosidase activity (Galacto-Star System; Applied Biosystems, Bedford, MA). A neutralization curve was generated by plotting the percentage of neutralization vs sCD4 dilution, and 50% inhibitory concentrations (IC_50_) were determined using the Prism 4 software (GraphPad, San Diego, CA). For assessment of coreceptor usage efficiency, 7 × 10^3^ TZM-bl cells per well of a 96-well plate were inoculated, in duplicates, with 2 ng p24 Gag antigen equivalent of the indicated pseudotyped virus in the absence or presence of 4-fold dilutions of the CCR5 antagonist PSC-RANTES. The cells were lysed after 72 hours at 37°C, processed for β-galactosidase activity, and IC_50_ determined using the Prism 4 software.

### Soluble gp120-CD4-Ig binding

To examine monomeric gp120-CD4 binding, gp120 glycoproteins from transfected 293T cells were metabolically radiolabeled for 48 hours with 100 μCi/mL [35S]-methionine/cysteine ([35S] protein labeling mix; Perkin-Elmer, Waltham, Mass) in Dulbecco's modified Eagle's medium lacking methionine and cysteine and supplemented with 5% dialyzed fetal bovine serum. Radiolabeled protein extracts were incubated with either a mixture of sera from HIV-1 infected individuals or CD4-Ig (a fusion protein in which the N-terminal two domains of CD4 are linked to the Fc component of immunoglobulin G [89]) in the presence of 70 μl of 10% Protein A-Sepharose (American BioSciences Inc, Boulder, CO) for 2 hr at 37ºC. The precipitates were analyzed on NuPAGE Novex Bis-Tris polyacrylamide gels (Invitrogen, Carlsbad, CA), followed by autoradiography and quantification with a PhosphorImager (Molecular Dynamics, Sunnyvale, CA).

### Neutralization assay

Virus neutralization was assessed using TZM-bl cells in 96-well plates. Briefly, equal volumes (50 μl) of pseudotyped viruses (2-3 ng p24 Gag equivalent) and 4-fold serial dilutions of IgG1b12, 443-52D and T20 were incubated for 1 h at 37°C and then added to cells, in duplicate wells, for an additional 2 hours at 37°C. 100 μl of medium was then added to each well and the virus-protein cultures maintained for 72 hours. Control cultures received virus in the absence of blocking agent. At the end of the culture period, the cells were lysed and processed for β-galactosidase activity. A neutralization curve was generated by plotting the percentage of neutralization vs agent dilution, and IC_50_ determined using the Prism 4 software.

### Statistical analysis

The Mann-Whitney U or unpaired T test was used to evaluate differences in susceptibility to sCD4, 1b12, 447-52D and T20; binding of gp120 to CD4-Ig; and infection of macrophages between the early (w2-4) and the evolving R5 viruses. These tests were also used to examine differences in cumulative viral load between RPs with and without coreceptor switch. P-values <0.05 were considered statistically significant.

## Competing interests

The authors declare that they have no competing interests.

## Authors’ contributions

KZ, AF, WH and CCM designed the study. KZ, AF, JT and A Frantzell carried out the experiments. KZ, AF, JS and CCM analyzed the results and drafted the manuscript. All authors read and approved the final manuscript.
